# Effect of ultrasound frequency on the Nakagami statistics of human liver tissues

**DOI:** 10.1371/journal.pone.0181789

**Published:** 2017-08-01

**Authors:** Po-Hsiang Tsui, Zhuhuang Zhou, Ying-Hsiu Lin, Chieh-Ming Hung, Shih-Jou Chung, Yung-Liang Wan

**Affiliations:** 1 Department of Medical Imaging and Radiological Sciences, College of Medicine, Chang Gung University, Taoyuan, Taiwan; 2 Medical Imaging Research Center, Institute for Radiological Research, Chang Gung University and Chang Gung Memorial Hospital at Linkou, Taoyuan, Taiwan; 3 Department of Medical Imaging and Intervention, Chang Gung Memorial Hospital at Linkou, Taoyuan, Taiwan; 4 Department of Electrical Engineering, Chang Gung University, Taoyuan, Taiwan; 5 College of Life Science and Bioengineering, Beijing University of Technology, Beijing, China; 6 Faculty of Information Technology, Beijing University of Technology, Beijing, China; The University of Hong Kong, HONG KONG

## Abstract

The analysis of the backscattered statistics using the Nakagami parameter is an emerging ultrasound technique for assessing hepatic steatosis and fibrosis. Previous studies indicated that the echo amplitude distribution of a normal liver follows the Rayleigh distribution (the Nakagami parameter *m* is close to 1). However, using different frequencies may change the backscattered statistics of normal livers. This study explored the frequency dependence of the backscattered statistics in human livers and then discussed the sources of ultrasound scattering in the liver. A total of 30 healthy participants were enrolled to undergo a standard care ultrasound examination on the liver, which is a natural model containing diffuse and coherent scatterers. The liver of each volunteer was scanned from the right intercostal view to obtain image raw data at different central frequencies ranging from 2 to 3.5 MHz. Phantoms with diffuse scatterers only were also made to perform ultrasound scanning using the same protocol for comparisons with clinical data. The Nakagami parameter–frequency correlation was evaluated using Pearson correlation analysis. The median and interquartile range of the Nakagami parameter obtained from livers was 1.00 (0.98–1.05) for 2 MHz, 0.93 (0.89–0.98) for 2.3 MHz, 0.87 (0.84–0.92) for 2.5 MHz, 0.82 (0.77–0.88) for 3.3 MHz, and 0.81 (0.76–0.88) for 3.5 MHz. The Nakagami parameter decreased with the increasing central frequency (*r* = −0.67, *p* < 0.0001). However, the effect of ultrasound frequency on the statistical distribution of the backscattered envelopes was not found in the phantom results (*r* = −0.147, *p* = 0.0727). The current results demonstrated that the backscattered statistics of normal livers is frequency-dependent. Moreover, the coherent scatterers may be the primary factor to dominate the frequency dependence of the backscattered statistics in a liver.

## Introduction

Liver fibrosis is a critical factor leading to cirrhosis and hepatocellular carcinoma [1]. Hepatitis steatosis may progress to nonalcoholic steatohepatitis, fibrosis, cirrhosis, and hepatocellular carcinoma [2]. Currently, biopsy is the gold standard for diagnosing liver fibrosis and fatty liver. Considering the invasiveness and sampling errors of liver biopsy [3,4], imaging-based assessments of hepatic steatosis and fibrosis are gaining scientific and medical interests for screening and characterizing liver diseases. Several studies have indicated magnetic resonance (MR) elastography as a reliable imaging method for evaluating hepatic fibrosis [5–7]. [^1^H]-MR spectroscopy is presently considered a standard method for the noninvasive evaluation of fatty livers because the obtained value correlates well with the histological fat content of a liver [8–10]. However, these MR-based approaches are expensive and time consuming, thus complicating clinically routine examinations.

Compared with MR techniques, ultrasound imaging provides real-time screening of the liver and can be used in clinically routine examinations of liver diseases. The imaging features of the liver in B-mode ultrasound are frequently used by physicians to evaluate liver diseases. The liver surface (smooth, irregular, or undulated), liver parenchyma (homogeneous, heterogeneous, or coarse), hepatic vessel (smooth, obscure, or narrow), and spleen size (<20 cm^2^ or >20 cm^2^) correlate well with the severity of liver fibrosis [11–13]. The liver–diaphragm differentiation in the echo amplitude, ultrasound penetration, and hepatic vessel clarity in B-mode imaging are associated with the fatty liver degree [14,15]. Notably, features and textures of B-mode images may be operator-dependent and vary with some system settings (e.g., gain, time gain compensation, and signal and image processing) [16]. Interpretations may vary among physicians. Therefore, the quantitative computational analysis of the raw image data (the beamformed radiofrequency [RF] signals backscattered from tissues) may facilitate a more objective evaluation of liver diseases.

A normal liver parenchyma may be typically considered a three-dimensional arrangement of considerable scatterers [17,18]. Under this condition, the statistics of the echo amplitude for normal liver tissues typically follows the Rayleigh distribution [19–22]. Recently, methods based on the analysis of the envelope statistics have gained high impetus and are introduced for evaluating diffuse liver diseases, including hepatic fibrosis [23–27] and fatty liver [28–30]. Using mathematical distributions to model the backscattered statistics is also a useful strategy for assessing liver diseases. In particular, the Nakagami distribution is the most frequently adopted model for envelope statistics analysis because of its simplicity and low computational complexity [31]. Ultrasound Nakagami parametric imaging on the basis of the Nakagami distribution has been proposed for characterizing scatterers in a scattering medium [32,33] and demonstrated to facilitate the visualization of changes in the echo amplitude distribution caused by liver fibrosis [34–36] and fatty liver [37,38].

Clinically, convex array transducers with 2–5-MHz bandwidths are typical probes used for screening livers. The transmitting central frequency and receiving bandwidth of the convex transducer can be practically adjusted for general fundamental or higher frequency imaging (e.g., tissue harmonic imaging) depending on the clinical requirements of image penetration and resolution [39,40]. Small-part linear array transducers, with central frequencies ranging from 5 to 12 MHz, also benefit clinical evaluations of liver diseases [41–43]. While using different ultrasound frequencies for liver imaging, the resolution cell size, which is determined by the pulse length and beamwidth, varies with frequency [44]. An increasing ultrasound frequency results in a smaller size of the resolution cell, reducing the number of scatterers in the resolution cell. Under this condition, the echo amplitude distribution of normal liver tissues may not continue following the Rayleigh distribution. For this reason, while the Rayleigh distribution is clinically used as a ground truth to interpret the statistical properties of the envelope signals from normal human livers [19–22], clarifying the frequency dependence of the backscattered statistics in human livers is highly required.

On the other hand, as we mentioned earlier, a liver parenchyma can be modeled as a collection of scatterers. Hepatocytes were proposed as scatterers in a liver to contribute diffuse scattering [45,46]. The connective tissue network was also considered as the source of ultrasound scattering in the liver [47]. A study suggested that major scatterers are the areas of veins, arteries, and ducts [48]. The two-component scattering model was used to explain that portal triads act as the source of coherent scattering [49,50]. The above literatures revealed that ultrasound scattering in a liver is contributed by both diffuse and coherent scatterers. However, there is a lack of experimental evidences to explain how the diffuse and coherent scatterers affect the frequency-dependent statistical properties of the envelope signals.

In this study, the backscattered signals of human livers (a natural two-component model containing diffuse and coherent scatterers) and agar phantoms with randomly located scatterers (diffuse scattering only) were acquired for B-mode and Nakagami imaging at different central frequencies. According to the experimental results, we aimed to discuss (i) the effect of ultrasound frequency on the envelope statistics of human livers and the corresponding Nakagami parameters; (ii) the sources of ultrasound scattering and the type of scatterers that dominate the frequency-dependent properties of the backscattered statistics in a liver.

## Theoretical background

### Statistical distributions

The Rayleigh distribution is the first model to describe the statistical distribution of ultrasound backscattered envelope signals, in case of a high density of randomly distributed scatterers without coherent signal components [51]. A more versatile model is the homodyned K-distribution, which can model tissues with low scatterer number densities and those with coherent signal components because of periodically located scatterers [52–55].

The Nakagami distribution is presented as an approximation of the homodyned K-distribution and is the most frequently adopted tissue characterization model because of its simplicity [31]. Shankar [56] introduced the Nakagami distribution for modeling the ultrasound backscattered statistics. The probability distribution function of the Nakagami model is determined using the following formula [56]:

$$f(R)=\frac{2m^{m}R^{2m-1}}{\Gamma (m)\Omega^{m}}\text{exp}\left(-\frac{m}{\Omega }R^{2}\right)U(R),$$ (1)

where *R* means the echo envelope signals; Γ(∙) and *U*(∙) are the gamma and unit step functions, respectively; *E*(∙) denotes the statistical mean. The scaling parameter Ω and the Nakagami parameter *m* associated with the Nakagami distribution can be respectively obtained using

$$\Omega =E(R^{2})$$ (2)

and

$$m=\frac{{\left[E(R^{2})\right]}^{2}}{E[R^{4}]-{\left[E(R^{2})\right]}^{2}}.$$ (3)

The Nakagami parameter is the shape parameter of the Nakagami distribution that allows classifying the envelope statistics according to its value: (i) *m* < 0.5, the distribution is considered Nakagami gamma (few scatterers with gamma-distributed scattering cross-sections in the resolution cell); (ii) 0.5 ≤ *m* ≤ 1, the backscattered statistics follows the pre-Rayleigh distribution (few or strong scatterers mixed with the randomly distributed scatterers in the resolution cell); (iii) *m* = 1, this value shows the Rayleigh distribution (a large number of randomly distributed scatterers in the resolution cell); (iv) *m* > 1, the backscattered statistics follows the post-Rayleigh distribution (both periodically located and randomly distributed scatterers in the resolution cell). The Nakagami parameter easily designates the type of the backscattered statistics. Therefore, Shankar [57] suggested ultrasound Nakagami imaging on the basis of a Nakagami parametric map for describing the envelope statistics. Currently, Nakagami imaging has been systematically developed for tissue characterization [32,33,58–62].

### Ultrasound Nakagami imaging

**Fig 1:**
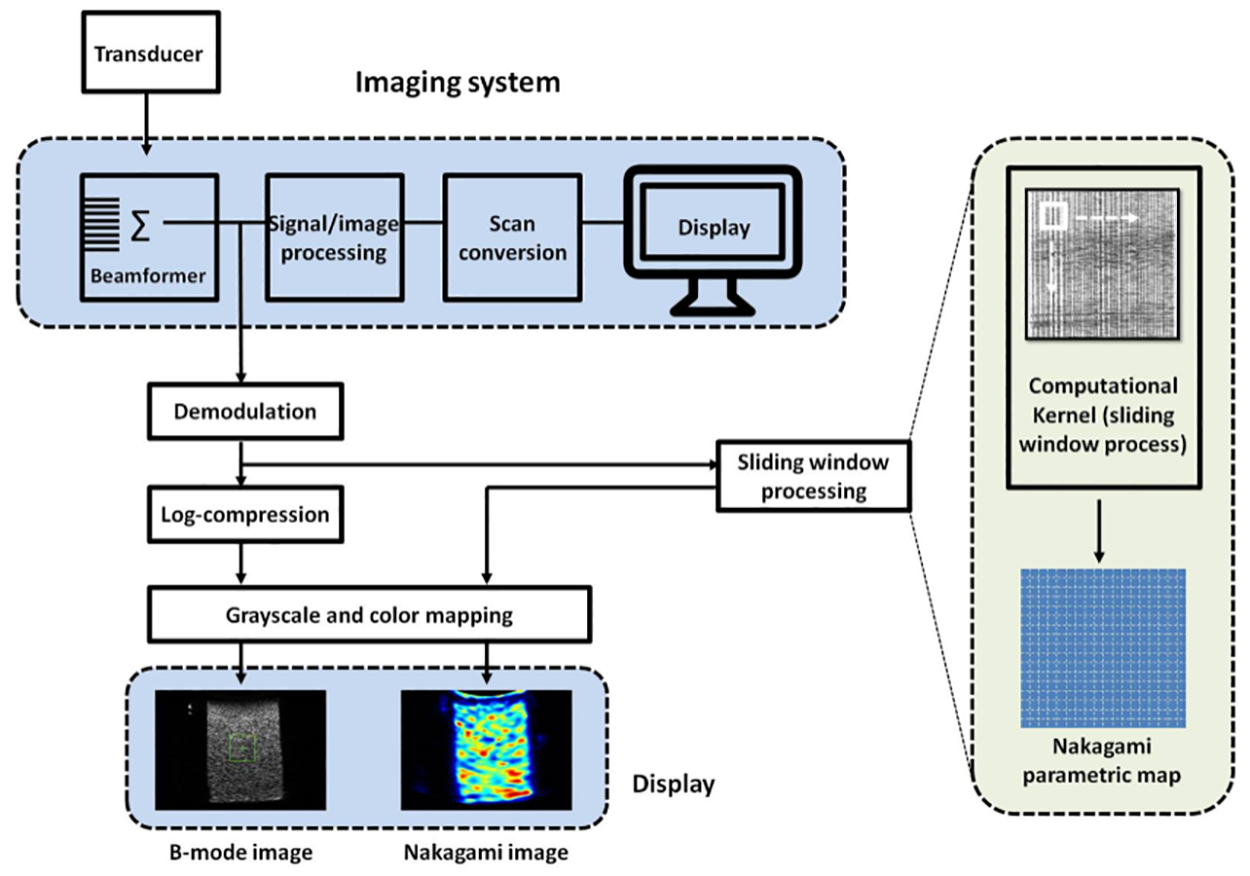
Flowchart for constructing a Nakagami image. The beamformed RF signals are demodulated to obtain envelope images, which are further processed using the sliding window technique to calculate local Nakagami parameters using Eq (3) for Nakagami parametric imaging.

In this study, ultrasound Nakagami imaging was used to image the backscattered statistics of human liver. In brief, (i) the envelope image (i.e., the echo amplitude data) was obtained using the absolute value of the Hilbert Transform of each echo signal; (ii) a square window within the envelope image was used to collect local amplitude data for estimating the Nakagami parameter using Eq (3), which is assigned as the new pixel located in the center of the window; (iii) the window was allowed to move throughout the envelope image in a one-pixel step, and step 2 was repeated to construct a Nakagami parameter map. Note that using small windows to acquire envelope data results in overestimation of the Nakagami parameter; whereas Nakagami parametric imaging based on large windows makes the resolution become lower. For this reason, using an appropriate size of the sliding window to construct a parametric image is necessary to yield stable parameter estimation and an acceptable resolution. According to previous studies, the minimally required side length of the sliding window used for constructing a Nakagami image is three times the pulse length of the transducer [32,33]. This study adopted the above criterion to determine the window size for ultrasound Nakagami imaging. Using a side length that is too long (i.e., a much larger window size) degrades the image resolution although it does not produce a significant effect on the parameter estimation [62]. The algorithmic procedure is shown in Fig 1.

## Materials and methods

### Imaging system

To explore the effects of ultrasound frequency on the backscattered statistics of human livers, the image data acquired at different central frequencies should correspond to the same scanning plane (i.e., the tissue section) for comparisons. However, the breaths and motions of participants during the examinations may result in that the acquired image data at different time points correspond to different tissue cross-sections. To reduce the uncertainties in imaging the same tissue section, a real-time data acquisition system is required in the experimental design.

**Fig 2:**
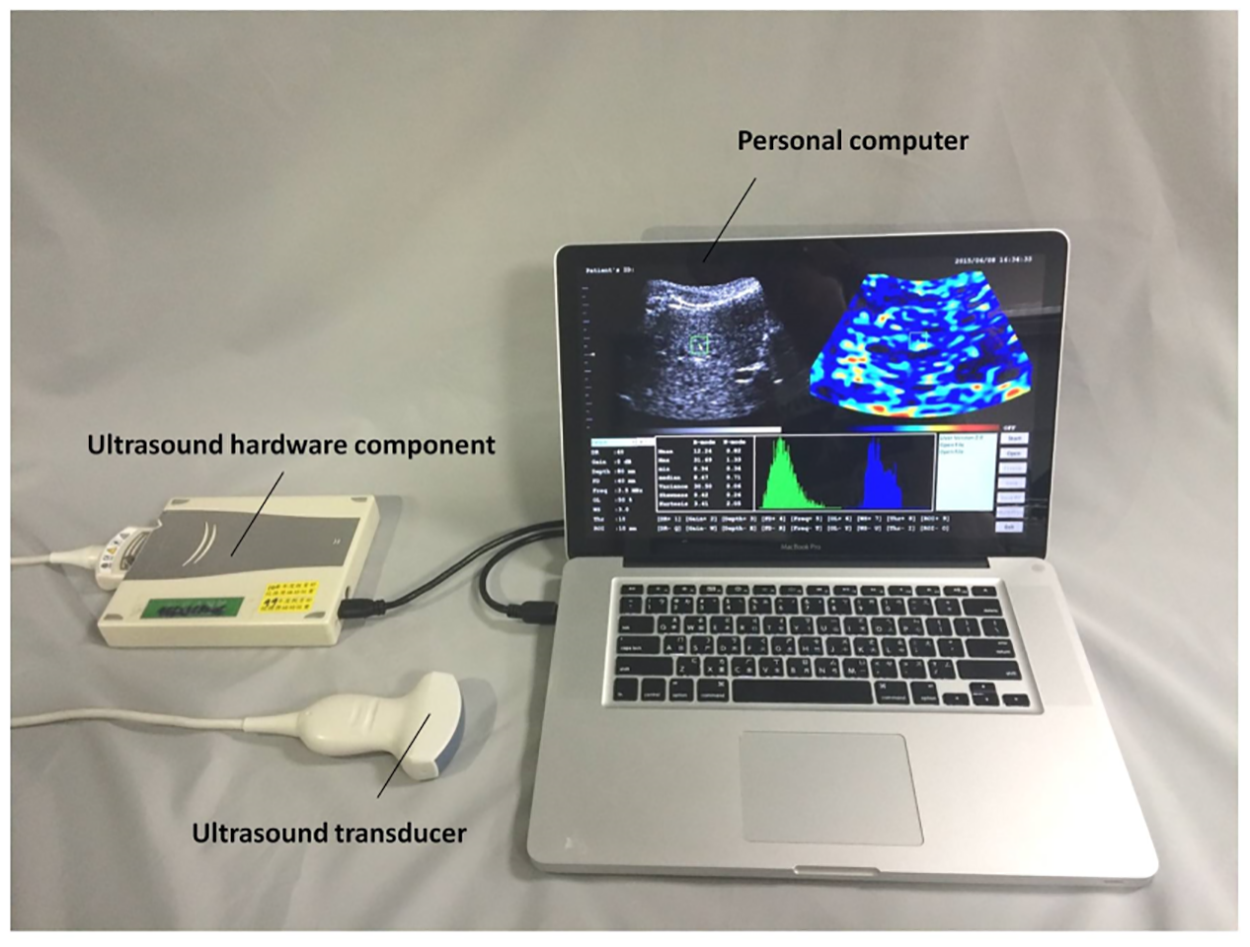
Images of the system and API. A portable clinical ultrasound scanner (Terason 3000) was used as the system platform for developing a real-time Nakagami imaging system. The software development kit provided by the manufacturer was used for system control, interface communication, and data access. A keyboard shortcut function was created to allow users to acquire image data using different central frequencies in one second.

A portable clinical ultrasound scanner (Model 3000, Terason, Burlington, MA, USA) was used as a system platform, as shown in Fig 2. The system comprises a convex transducer with 128 elements (Model 5C2A, Terason) and a hardware module connected to a computer (MacBook Pro laptop operating on Windows XP environment; CPU: 2.5 GHz dual-core Intel Core i5 processor) through an IEEE 1394 interface. The software development kit (SDK) provided by the manufacturer was used for system control, central frequency selection (2, 2.3, 2.5, 3, and 3.5 MHz), interface communication, and data access. An application programming interface (API) was designed to integrate the SDK and Nakagami imaging algorithm, as described in the Theoretical Background section, on the system by using C++ with OpenCV 2.4.3. During API application, image beamformed RF data after signal digitalization were directly accessed from the buffer for real-time ultrasound Nakagami imaging and parameter analysis. The characteristics of the system are summarized in Table 1. The imaging settings can be adjusted using the API, and the system enables a real-time display of B-mode and Nakagami images with a maximum frame rate of 20 frames/s. A square region of interest (ROI) can be selected on the images for real-time parameter analysis. All analysis results were immediately shown on the interface. To avoid measurement biases in imaging the same tissue section, a keyboard shortcut function was created to allow users to acquire image data at different central frequencies from 2 to 3.5 MHz in one second.

**Table 1:** Pulse lengths, beam widths, and sizes of the resolution cell at different central frequencies ranging from 2 to 3.5 MHz. Pulse lengths were measured from two-way pulse-echo testing of the transducer. Beam widths were evaluated from the widths of the lateral distributions (measured at 20% of the maximum) of the B-mode intensity corresponding to the point targets (diameters: 0.1 mm) in a commercial phantom (Model 054GS, CIRS, Norfolk, Virginia, USA). For each central frequency, five measurements were carried out at the focal lengths of 1 cm and 4 cm, respectively (left: data measured at 1-cm focus; right: data measured at 4-cm focus).

Frequency (MHz)	Pulse length (cm)	Beam width (cm)	Size of the resolution cell (mm^3^)
**3.5**	0.18±0.03 / 0.19±0.01	0.24±0.02 / 0.24±0.02	8.14 / 8.59
**3.3**	0.20±0.02 / 0.20±0.02	0.25±0.03 / 0.26±0.02	9.81 / 10.62
**2.5**	0.21±0.03 / 0.22±0.02	0.27±0.04 / 0.27±0.05	12.02 / 12.60
**2.3**	0.22±0.02 / 0.23±0.01	0.39±0.02 / 0.38±0.03	26.26 / 26.08
**2**	0.23±0.03 / 0.23±0.03	0.45±0.03 / 0.47±0.04	36.56 / 39.90

Note—Shorter pulse lengths and beam widths result in better axial and lateral resolutions, respectively. Assuming that the resolution cell of the transducer is a cylinder, the sizes of the resolution cell were calculated using the average pulse lengths and beam widths.

### Clinical measurements

A total of 30 healthy participants were enrolled in Chang Gung University between August 2014 and July 2015. This study was approved by the Institutional Review Board of Chang Gung Memorial Hospital. All participants signed informed consent forms, and all the experiments were performed in accordance with the approved guidelines. The participants had none of the following conditions: drinking, smoking habits, remarkable past medical history, and clinical symptoms and signs of liver or renal parenchymal diseases. Before measurements, demographic information (including age, sex, and body mass index) were recorded for each patient, as shown in Table 2.

**Table 2:** Characteristics of the study participants. The participants had none of the following conditions: drinking, smoking habits, remarkable past medical history, and clinical symptoms and signs of liver or renal parenchymal diseases.

Characteristics	Value
**Male/Female**	20/10 (*n* = 30)
**Age, years**	
Mean ± standard deviation (range)	22.3 ± 1.29 (20–26)
Median	22
**BMI, kg/m**^**2**^	
Mean ± standard deviation (range)	22.98 ± 5.26 (15.05–41.50)
Median	20.90

Note—Unless otherwise noted, data are numbers of patients. BMI: body mass index. BMI was calculated and defined according the Department of Health in Taiwan: optimal BMI was defined as 18.5 ≦ BMI < 24 kg/m^2^, overweight as 24 ≦ BMI < 27 kg/m^2^, and obesity was defined as BMI ≧ 27 kg/m^2^.

**Fig 3:**
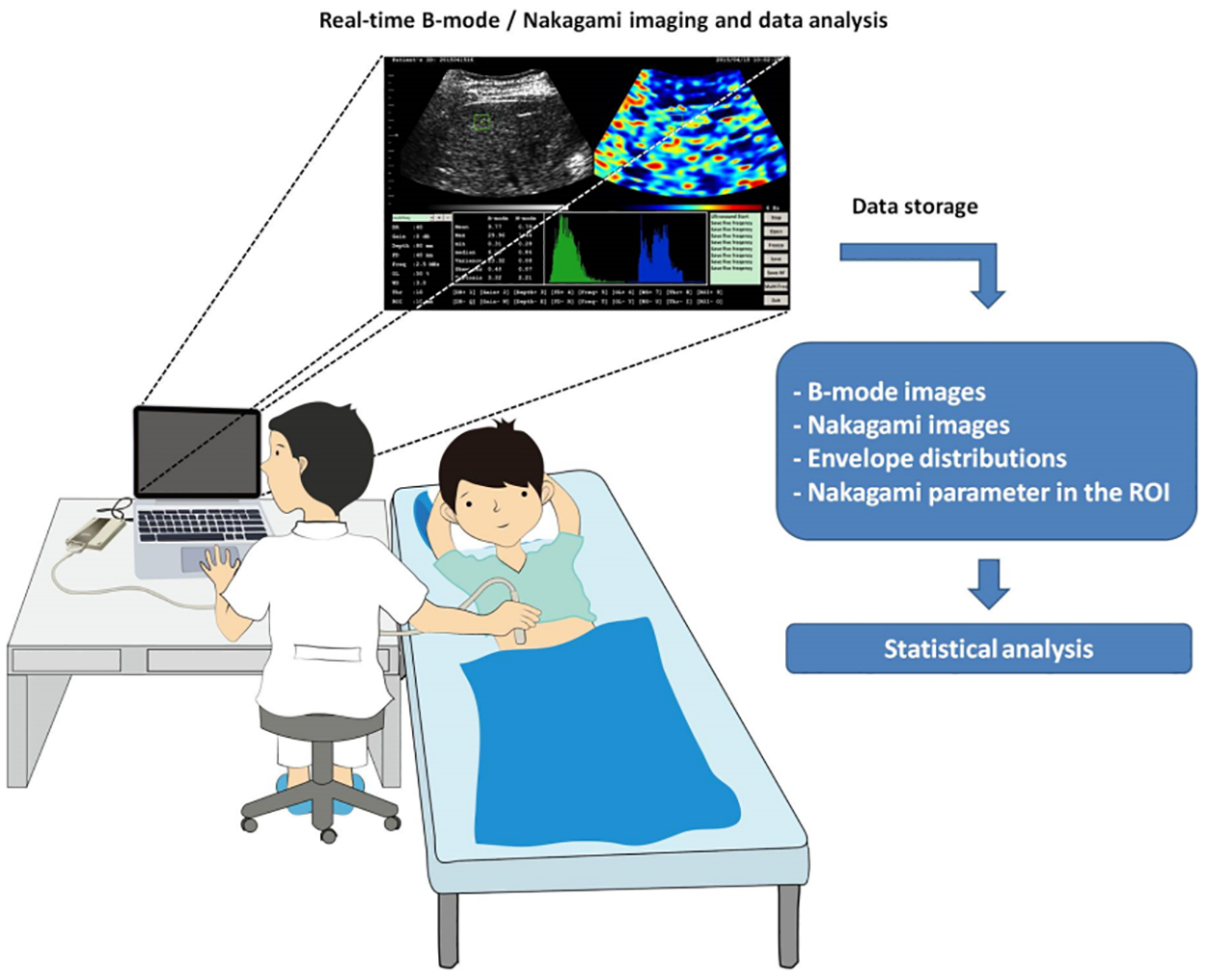
All participants underwent a standard care ultrasound examination. A radiologist scanned the liver of each participant from the right intercostal view. All images and analysis results were immediately shown on the interface. After scanning, multiple frequency data were saved and used for statistical analysis.

A radiologist, who was blind to the hypothesis of the study, scanned the liver of each participant from the right intercostal view (Fig 3). The absence of acoustic shadowing artifacts and exclusion of large vessels in the region of analysis were the criteria for successful scanning. The imaging focus and depth were set at 4 and 8 cm, respectively. In a previous study [38], some basic criteria were suggested to select the ROI for liver image analysis: (i) using a relatively small ROI to locate on the liver parenchyma. A small ROI easily excludes blood vessels (e.g., portal venous branches or hepatic veins) to reduce the bias of analyzing liver parenchyma; (ii) the location of the ROI should be at the focal zone, reducing the effects of attenuation and diffraction on the image analysis; (iii) using multiple ROIs in a single image for analysis may increase the probability of incursion by vessels. Alternatively, selecting one ROI in each single image obtained from multiple scans of the liver and averaging the results in each ROI are suggested. Therefore, during each scanning, an ROI of 1 × 1 cm located at the depth of the focal zone was marked on the B-mode image of the liver parenchyma to reduce the effect of attenuation. Concurrently, the ROI was also automatically duplicated by the software to appear at the same location on the corresponding Nakagami image. The B-mode and Nakagami images at different central frequencies were acquired by using the keyboard shortcut function. Five scans were performed for each participant. The average of the Nakagami parameters in the ROI was used as an estimate of the ultrasound backscattered statistics.

### Phantom experiments

Experiments on phantoms containing diffuse scatterers only were carried out to explore the frequency dependence of the envelope statistics in a condition without any coherent scatterers.

Six phantoms were constructed by boiling and cooling agar–water mixtures (dissolving 3 g of the agar powder into 200 mL of water) and adding 2 g graphite powder with diameters <20 μm (Model 282863, Sigma-Aldrich, St. Louis, MO, USA). The number densities of scatterers (NDS) of the phantoms can be estimated by

$$\text{NDS}=\frac{M}{\frac{4}{3}\pi \;r_{g}{}^{3}\cdot \rho \cdot V},$$ (4)

where *M*, *r*_*g*_, and *ρ* correspond to the mass, radius, and density of the scatterers, respectively, and *V* denotes the volume of the agar phantom. A large amount of graphite powder was added for the formation of the backscattered signals because graphite powders with small diameters (<20 μm) are relatively weak scatterers. Using Eq (4) and assuming that the average diameter of graphite powder is 20 μm, the number density of scatterers in the background of the phantom was estimated to be at least 1000 scatterers/mm^3^. This estimated number density ensures that a large number of randomly distributed scatterers exist in the resolution cell, making backscattered envelopes obey the Rayleigh distribution. However, while adding a large amount of graphite powder to contribute backscattered signals, it also results in the attenuation effect, limiting the penetration depth of acoustic waves. Consequently, the data acquisition and analysis procedures were the same as those used in the clinical measurements, except that the imaging focus and depth were set at 1 and 2 cm, respectively. Note that no significant differences of the transducer characteristics between 1-cm (for phantom scanning) and 4-cm (for human liver scanning) focuses were observed, as shown in Table 1. The B-mode and Nakagami images at different central frequencies were acquired, and a ROI of 1 × 1 cm located at the focal zone was used to calculate the average Nakagami parameter of each Nakagami image. For each phantom, five independent scans were performed so that a total of 30 measurements of the Nakagami parameter were used for the statistical analysis.

### Statistical analysis

The Nakagami parameter as a function of ultrasound central frequency was expressed as the median and interquartile range (IQR). The Pearson correlation coefficient *r* and probability value *p* were calculated for evaluating the Nakagami parameter–frequency correlation. All statistical analyses were performed using SigmaPlot software (Version 12.0, Systat Software, Inc., CA, USA).

## Results

**Fig 4:**
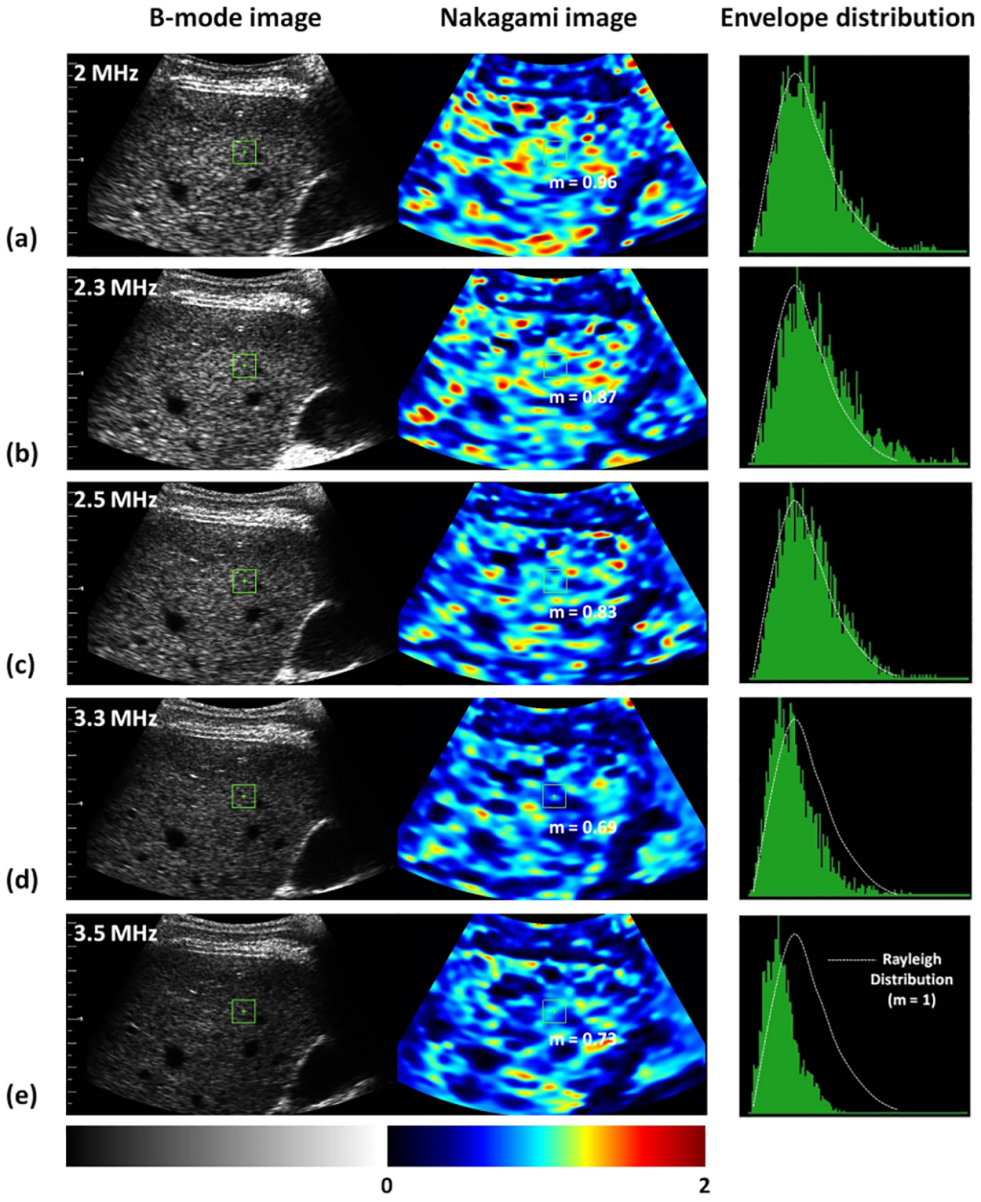
Typical B-scan, Nakagami images, and the envelope distributions of liver tissues obtained at ultrasound central frequencies ranging from 2 to 3.5 MHz. The envelope distributions were expressed using the histograms (plotted with 100 green bins). The white dotted lines mean the Rayleigh distribution. The shading of the Nakagami images gradually varied toward blue with the increasing ultrasound central frequency, corresponding to changes in the echo amplitude distribution of the signals acquired in the ROI to the pre-Rayleigh distribution.

**Fig 5:**
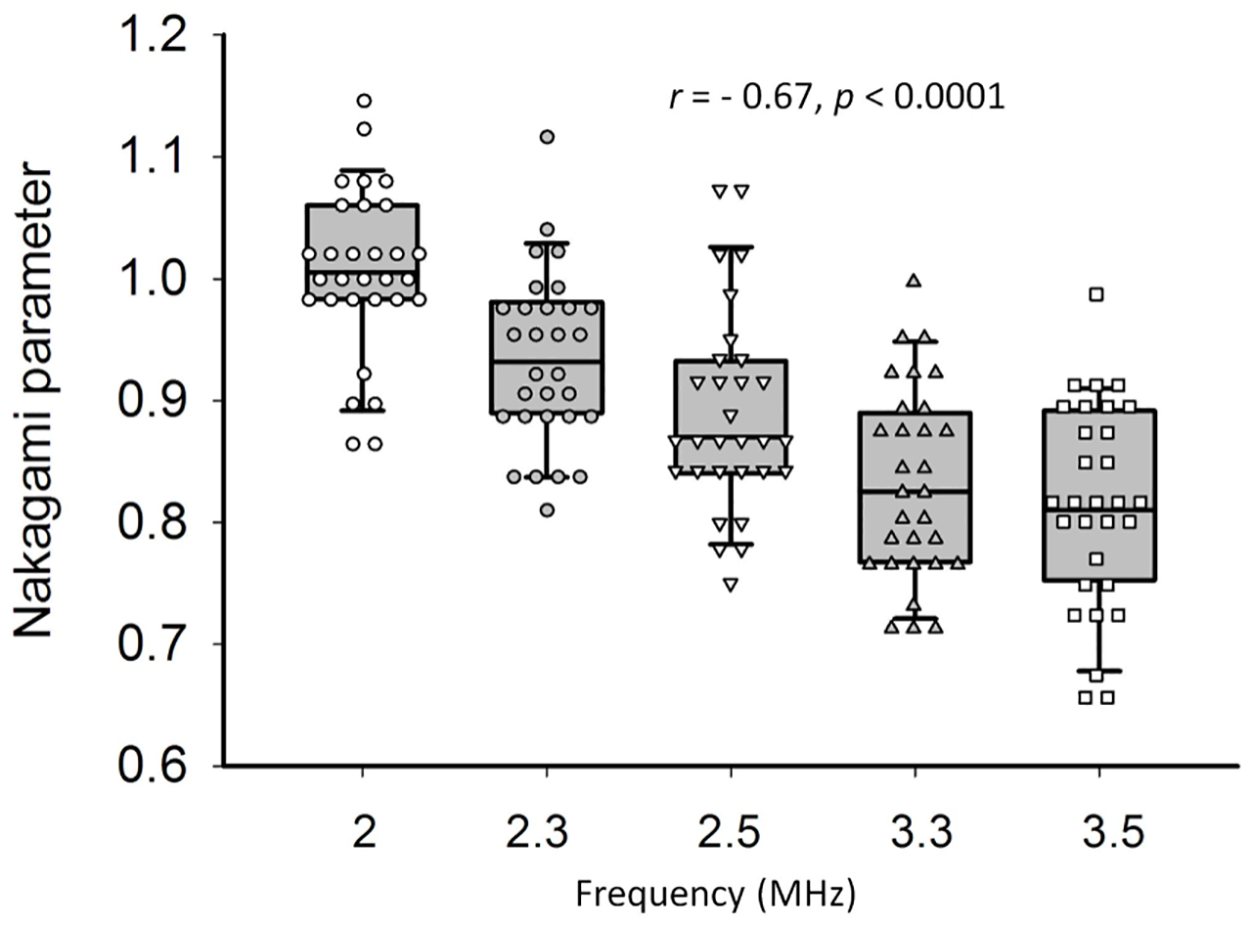
Nakagami parameter of the human liver as a function of central frequency. Data were expressed using the box plot. The median Nakagami parameter was 1.00 (IQR: 0.98–1.05) for 2 MHz, 0.93 (IQR: 0.89–0.98) for 2.3 MHz, 0.87 (IQR: 0.84–0.92) for 2.5 MHz, 0.82 (IQR: 0.77–0.88) for 3.3 MHz, and 0.81 (IQR: 0.76–0.88) for 3.5 MHz. The Nakagami parameter consistently decreased with the increasing central frequency (*r* = −0.67, *p* < 0.0001).

Fig 4 shows the typical images (including B-mode and Nakagami images) and the envelope distributions of liver tissues at ultrasound central frequencies of 2–3.5 MHz. The shading of the Nakagami images gradually varied toward blue with the increasing ultrasound central frequency. Concurrently, the envelope distribution was gradually far away from the Rayleigh distribution, corresponding to changes in the echo amplitude distribution of the signals acquired in the ROI to the pre-Rayleigh distribution. Fig 5 shows the Nakagami parameters obtained from livers corresponding to each central frequency. The Nakagami parameter varied from 0.65 to 1.15. The median Nakagami parameter was 1.00 (IQR: 0.98–1.05) for 2 MHz, 0.93 (IQR: 0.89–0.98) for 2.3 MHz, 0.87 (IQR: 0.84–0.92) for 2.5 MHz, 0.82 (IQR: 0.77–0.88) for 3.3 MHz, and 0.81 (IQR: 0.76–0.88) for 3.5 MHz. The Nakagami parameter of the human liver consistently decreased with the increasing central frequency (*r* = −0.67, *p* < 0.0001).

**Fig 6:**
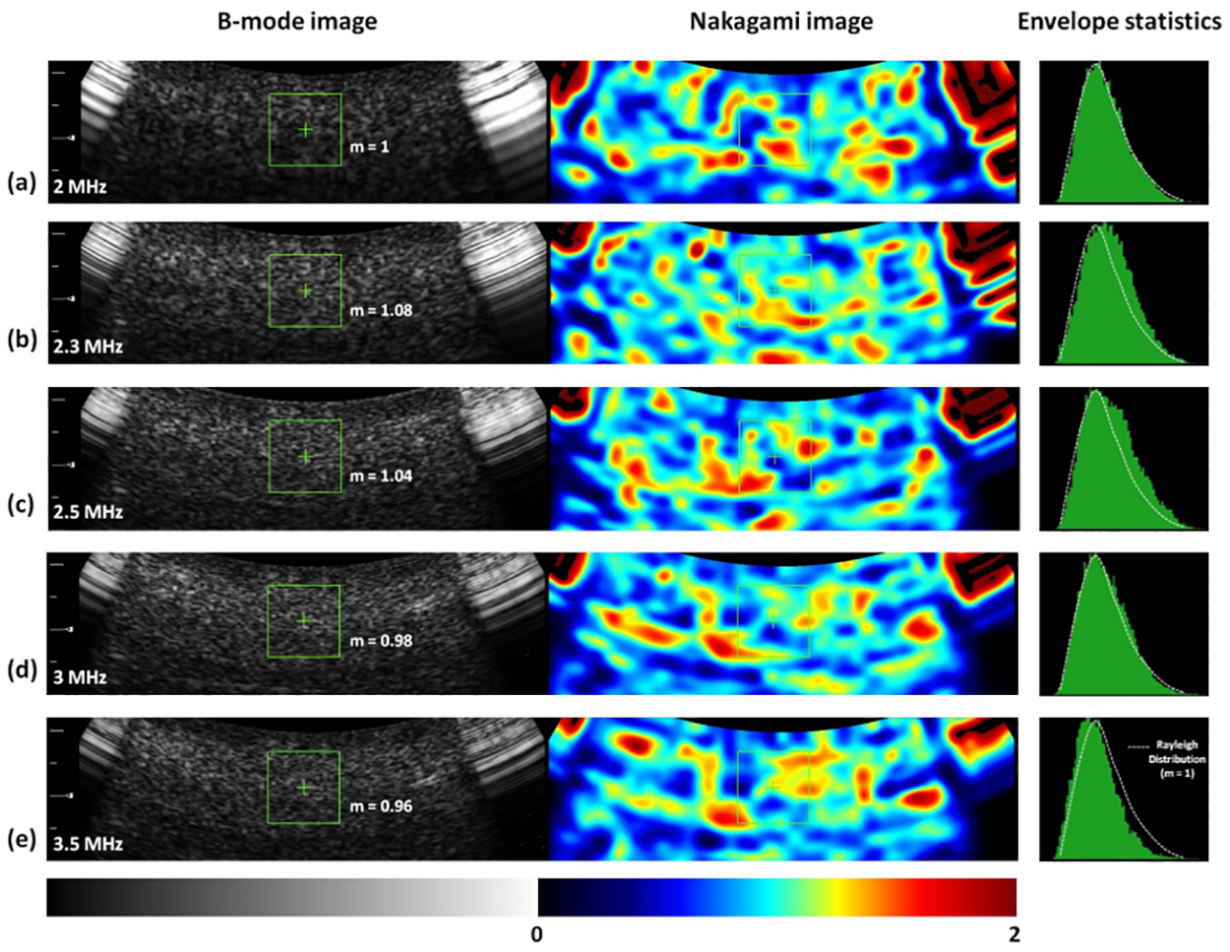
Typical B-scan, Nakagami images, and the envelope distributions (100 bins; white dotted lines mean the Rayleigh distribution) of the phantoms obtained using the central frequencies ranging from 2 to 3.5 MHz. The shading of the Nakagami images did not change significantly with increasing the central frequency. The envelope statistics at different central frequencies were close to the Rayleigh distribution.

**Fig 7:**
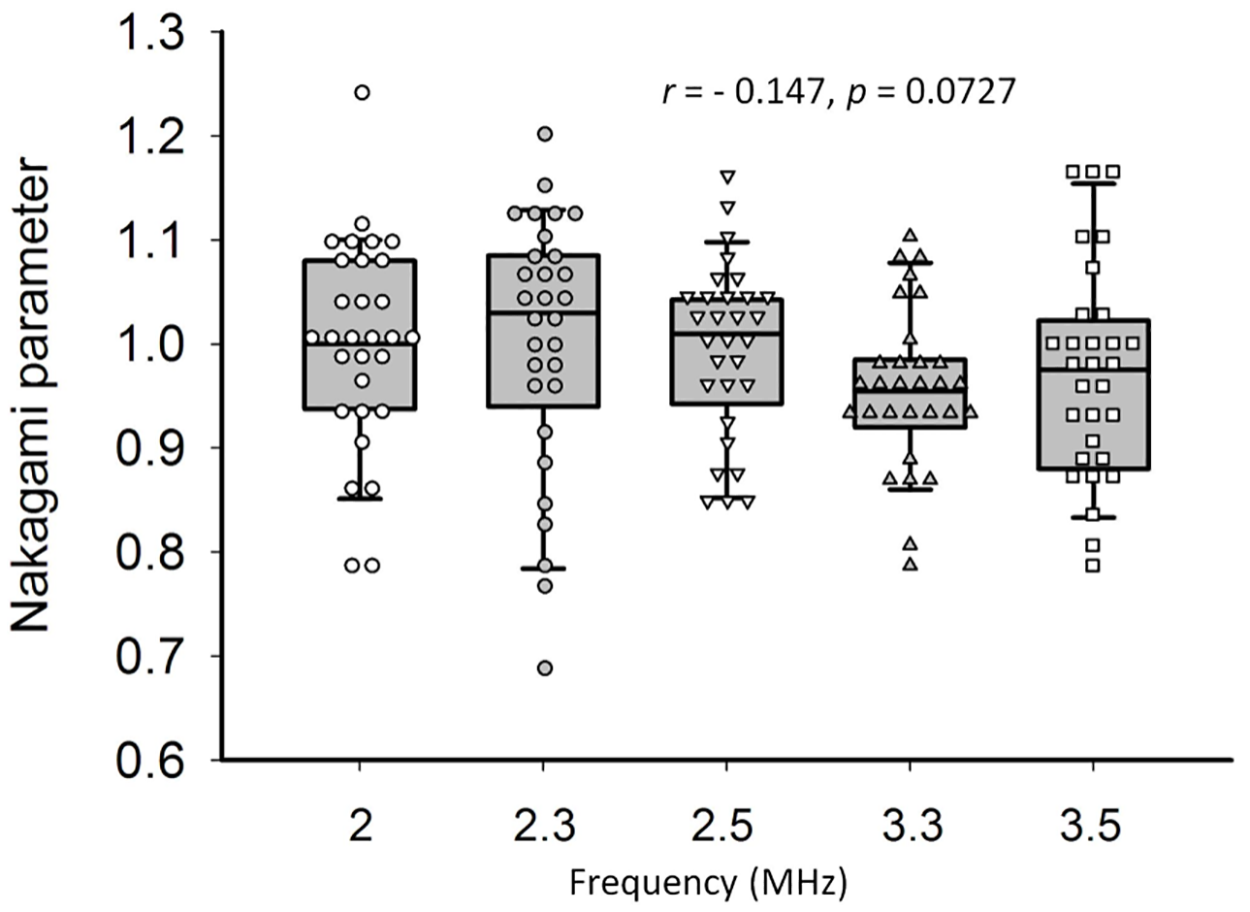
The median Nakagami parameter was 1.00 (IQR: 0.94–1.07) for 2 MHz, 1.03 (IQR: 0.95–1.08) for 2.3 MHz, 1.01 (IQR: 0.95–1.04) for 2.5 MHz, 0.96 (IQR: 0.92–0.98) for 3.3 MHz, and 0.97 (IQR: 0.89–1.01) for 3.5 MHz. The Nakagami parameter of the phantom was uncorrelated with the central frequency (*r* = −0.147, *p* = 0.0727).

Fig 6 shows the B-scans, Nakagami images, and the envelope distributions obtained from the phantoms using central frequencies of 2–3.5 MHz. The shading of the Nakagami images did not change significantly with increasing the central frequency, and the envelope statistics at different central frequencies were close to the Rayleigh distribution. Fig 7 shows the Nakagami parameters obtained from the phantoms as a function of central frequency. The median Nakagami parameter was 1.00 (IQR: 0.94–1.07) for 2 MHz, 1.03 (IQR: 0.95–1.08) for 2.3 MHz, 1.01 (IQR: 0.95–1.04) for 2.5 MHz, 0.96 (IQR: 0.92–0.98) for 3.3 MHz, and 0.97 (IQR: 0.89–1.01) for 3.5 MHz. The Nakagami parameter of the phantom was uncorrelated with the central frequency (*r* = −0.147, *p* = 0.0727).

## Discussion

### Significance of this study

The current findings indicated that the backscattered statistics and the corresponding Nakagami parameter of human liver tissues are frequency-dependent. Significant frequency dependences of the backscattered statistics and the Nakagami parameter were not observed in the measurements of the phantoms. The above two experimental evidences supported the following suggestions: (i) conventionally, the Rayleigh distribution is well accepted as the behavior of the backscattered statistics for human livers [19–22]. Now, it has been shown that the envelope statistics of human livers is not necessarily the Rayleigh distribution, depending on the use of the frequency. A previous study (using 5-MHz ultrasound for liver scans) [30] reported that the backscattered statistics of normal human livers follow pre-Rayleigh distributions because of the presence of small vessel walls. Besides the vessel wall effects, the frequency that affects the size of the resolution cell also plays an important role in dominating the formation of pre-Rayleigh statistics of normal livers, as supported by the current experimental findings. A modified viewpoint based on the non-Rayleigh or the frequency-dependent backscattered statistics should be adopted as a more generalized explanation for the statistical properties of the backscattered signals returned from human livers; (ii) compared with the phantoms with diffuse scatterers only, the human liver is a natural two-component model containing diffuse and coherent scatterers. Thus, based on comparisons of Figs 5 and 7, the coherent scatterers may be the key factors to dominate the frequency dependence of the backscattered statistics. A previous study also showed that coherent scatterers may result in the Nakagami parameters smaller than 1, depending on the spacing of periodically located structures [56]. In the next sections, possible sources of ultrasound scattering in a liver are discussed.

### Sources of diffuse scattering in a liver

Diffuse scatterers may be defined as objects with dimensions smaller than the wavelength to make the echo amplitude and the phase differences insensitive to the orientation of the object [63]. Cells may be treated as the typical diffuse scatterers in a tissue. Biological tissues comprise of cells interconnected to perform a similar function within an organism. Cells are the basic structural, functional, and biological unit of all living organisms; therefore, many studies have focused on the interaction between the ultrasound wave and the cells to explore the properties of ultrasound scattering [64–67]. A hepatocyte is a cell of the main parenchymal tissue of the liver; it is approximately cubical, with side lengths of 20–30 μm and volume of 3.4 × 10^−6^ mm^3^ [68]. Note that when the resolution cell of the transducer contains a large number of randomly distributed scatterers (≥10) [19,69,70], the envelope of the backscattered signals (i.e., the echo amplitude) follows the Rayleigh distribution. Hepatocytes are very small; therefore, their number in the resolution cell can be >10 to fulfill the criterion for the Rayleigh distribution despite the ultrasound frequency used for driving the transducer. This is why hepatocytes were explained to act as scattering sources at the Rayleigh scattering level [46]. Studies have reported hepatic cells as scatterers in a liver that contribute to ultrasound scattering [45,46]. Many small vessels are only a few tenths of a millimeter in diameter and are also potential sites of diffuse scattering events and sources of ultrasonic speckle [46].

### Sources of coherent scattering and effective scatterers in a liver

Several literatures indicated that the portal triads act as the source of ultrasound coherent scattering in a liver [49,50,71,72]. In general, there are three to six portal triads per liver lobule, which is a polygonal mass of tissue composed of a central vein surrounded by plates and hepatocytes [46]. The portal triad located at the corners of the lobule contains a bile duct, a portal venule, a portal arteriole, and lymphatic vessels. All of these structures are surrounded by a sheath of connective tissue. It should be noted that the connective tissue network [47], veins, arteries, ducts [48], and hepatocytes [45,46] are able to contribute ultrasound backscattered signals. Therefore, the liver lobule not only are the structural and functional unit of a liver [73,74] but may also be considered as the effective scattering unit [31] that simultaneously involves diffuse and coherent scatterers for interpreting the interaction between ultrasound wave and the liver tissue.

As shown in Table 1, increasing the central frequency of the transducer from 2 to 3.5 MHz reduced the size of the resolution cell from approximately 40 to 8.6 mm^3^. The hexagonal diameter of liver lobules is approximately 1–1.5 mm, and the lobule length is 1.5–2 mm [48]. Approximating the shape of the liver lobule to a cylinder, the volume of the liver lobule is estimated to be 1.18–3.53 mm^3^, corresponding to an average of 2.36 mm^3^. Thus, the resolution cell of the transducer was expected to include 16.91, 11.05, 5.34, 4.50, and 3.64 effective scatterers (liver lobules), corresponding to ultrasound central frequencies of 2–3.5 MHz. Note that the elevational beam width is typically much larger than the axial and lateral directions. Thus, the size of the resolution cell may be underestimated, and the aforementioned estimated numbers of scatterers per resolution cell may not be highly precise; however, they provided the possibility revealing that <10 scatterers may exist in the resolution cell at higher frequencies to reduce the size of the resolution cell, as supported by the results of the average Nakagami parameter < 1 in Fig 5. The above discussion implies that the effective scattering model based on liver lobules may be a more appropriate consideration for describing the acoustic structures of human livers.

### Potential of ultrasound backscattered statistics in liver disease assessment

Besides ultrasound Nakagami imaging, the acoustic structure quantification (ASQ) technique has also recently gained attention as a tool for tissue characterization [23–25]. In ASQ, a focal disturbance (FD) ratio is defined from the areas under the histograms of two statistical parameters calculated using the average and variance of the echo signal amplitude. FD ratio is zero when the backscattered statistics obey the Rayleigh distribution. With increasing the degree of deviation from the Rayleigh distribution, the FD ratio becomes larger. The diagnostic value of the ASQ in liver characterization has been reported in several literatures [26–30]. Ultrasound Nakagami imaging or other statistical model-based parametric images may complement the ASQ for identifying the type of the backscattered statistics (pre-Rayleigh, Rayleigh, and post-Rayleigh) more precisely.

Ultrasound backscattered statistics has potential in the computer-assisted diagnosis of liver parenchymal diseases. Currently, ultrasound elastography is widely used as the ultrasound-based diagnostic method for evaluating liver parenchymal diseases because both fibrosis and steatosis alter liver elasticity (stiffness) [1,75]. Nevertheless, hepatic inflammation influences the estimation of liver stiffness [76–79] that causes uncertainties in measurements obtained through ultrasound elastography. Compared with ultrasound elastography, ultrasound backscattered statistics analysis reported no correlation with the blood alanine aminotransferase level [26]. Huang et al. [26] highlighted the relevance of backscattered statistics-based methods in the future assessment of liver fibrosis, particularly for patients with chronic hepatitis B. Moreover, compared with the B-scan, the Nakagami image was found to be more tolerant of the attenuation effect because the Nakagami parameter mainly depends on the statistical distribution of the envelope and less influenced by the overall signal magnitude [80]. The backscattered signals just need a relatively low SNR (11 dB) to endow the Nakagami parameter with the ability for characterizing the properties of tissues [81].

## Conclusions

This study has explored the effect of ultrasound frequency on the envelope statistics based on the Nakagami model and discussed the sources of ultrasound scattering in a human liver. The present work demonstrated the frequency dependence of the backscattered statistics of the liver tissues. With increasing the central frequency from 2 to 3.5 MHz, the envelope statistics of livers vary from the Rayleigh (the Nakagami parameter *m ≈* 1) to the pre-Rayleigh distribution (*m* < 1). Compared with the results of human livers, the effect of frequency on the backscattered statistics was not found in the phantoms with diffuse scatterers only. Therefore, the current findings and comparisons suggested: (i) the frequency-dependent backscattered statistics may be more appropriate than the conventional viewpoint of the Rayleigh statistics to describe the statistical properties of human livers in practice; (ii) the liver lobules, which were composed of the central vein, plates, connective tissues, hepatic cells (the dominant diffuse scatterers), and the portal triads (the coherent scatterers), may be considered as effective scatterers for ultrasound scattering in a liver. In particular, the coherent scatterers may dominate the frequency dependence of the backscattered statistics.

## Supporting information

S1 Data Data for Nakagami parameters as a function of central frequency obtained from livers and diffuse phantoms, respectively. (XLSX)
